# Walking energy harvesting and self-powered tracking system based on triboelectric nanogenerators

**DOI:** 10.3762/bjnano.11.141

**Published:** 2020-10-20

**Authors:** Mingliang Yao, Guangzhong Xie, Qichen Gong, Yuanjie Su

**Affiliations:** 1 State Key Laboratory of Electronic Thin Films and Integrated Devices, School of Optoelectronic Science and Engineering, University of Electronic Science and Technology of China (UESTC), Chengdu 610054, PR China

**Keywords:** harvesting walking energy, internet of things, mechanical energy, pedestrian flow area, self-powered tracking system, triboelectric nanogenerator

## Abstract

Due to the extensive energy consumption and high population density in modern cities, the collection and use of scattered walking energy from the stream of people is crucial for the development of a green ecological city. Herein, a flexible undulated electrode-based triboelectric nanogenerator (u-TENG) was integrated to the floor to scavenge walking energy from pedestrians, promoting the ordered collection of disordered and scattered energy. Driven by the steps of human walking, the output of the as-fabricated u-TENG are an open-circuit voltage of 86 V and a short-circuit current of 6.2 μA, which are able to continuously light up 110 light-emitting diode bulbs. In addition, a self-powered location-tracking system was prepared for pedestrian volume counting and passenger tracing with the purpose of reducing energy consumption in public areas. The proposed walking energy harvesting device is flexible, feasible, and unaffected by season, climate, or location. This work not only proposes a strategy for mechanical energy harvesting in public areas, including subway stations, hospitals, shopping malls, and business streets, but also offers a novel solution for smart cities and low-carbon transportation alternatives.

## Introduction

With the fast progress in urbanization and commercialization, energy acquisition for powering wearable electronics [1–5] and wireless sensor networks is in high demand. Mechanical energy, which is widely distributed in the environment, is one of the most general power sources. The human body is a rich source of mechanical energy [6]. Muscle stretching, for example, converts biochemical energy into mechanical motion with a peak efficiency of ≈25%, which can easily deliver an output power of more than 100 W [7]. Even though the population maintains a sustainable growth, there is a high concentration of people in almost every public area. However, this universal and widespread form of energy has been neglected and wasted daily.

So far, in order to harvest the energy resulting from human motion, several mechanical energy scavenging methods have been invented, including devices based on electrostatic [8–9], electromagnetic [10–11], and piezoelectric effects [12–14]. Since the output power density of an electromagnetic generator (EMG) is proportional to the square of the frequency, it is not very efficient for an EMG to harvest low-frequency human motions, especially if they are below 5 Hz [15]. With respect to the piezoelectric generator, the relatively low-energy conversion efficiency and the fabrication complexity greatly hinder its potential application in large-scale energy conversion systems. Consequently, a small, lightweight, highly efficient, and cost-effective device for collecting disordered and scattered mechanical energy from the crowds in public locations is urgently needed.

Recently, triboelectric nanogenerators (TENGs) have been invented, which offer an innovative combination between electrostatic induction and contact electrification. These devices are able to harvest mechanical energy from a vast array of sources, such as body motion [16–19], vibration [20–23], rotation [24–27], sound wave [28], air flow [29–31], water wave, and rain drops [32–34]. Furthermore, since they are able to convert mechanical motion into electrical energy, TENGs have been widely used to successfully construct reliable self-powered sensing systems with an excellent performance, which can be used as motion [35–37] and temperature sensors [38–39], UV detectors [40], tactile sensors [41–43], sensors for healthcare [44–47], humidity sensors, and gas sensors [48–51], for example.

In this work, a flexible undulated electrode-based triboelectric nanogenerator (u-TENG) was proposed and fabricated to scavenge the walking energy from areas with a high pedestrian flow. The as-prepared u-TENG is composed of two copper-coated nanostructured poly(tetrafluoroethylene) (PTFE) thin films as the back electrodes and an elastic undulated electrode in between. The undulated electrode serves as a spacer and also as an induction electrode for energy collection. Triggered by the steps of human walking, the open-circuit voltage and short-circuit current reach values up to 86 V and 6.2 μA, respectively, which is sufficient to light up 110 light-emitting diode (LED) bulbs. In addition, by integrating the u-TENGs with six electrode channels, a self-powered location-tracking system was developed. This walking energy harvesting device is flexible, durable, and feasible, regardless of the time of the day, season, climate, and weather, which makes it an ideal candidate for harvesting walking energy from a high-flow pedestrian area. Although an undulated electrode with similar shape design and working mechanism has been proposed and demonstrated in our previous work [34], in which it was used to collect the impact from water waves, it has been used for the first time in this current work to build up a self-powered location-tracking system and to harvest mechanical energy from human walking. This work unravels the practicality of the u-TENG, which can be used as a device for harvesting energy from human motion, as a self-powered tracking system, for transportation control, and for environmental monitoring.

## Experimental

### Surface modification of a PTFE film

The surface modification of a PTFE film was performed in a similar manner as described previously [34]. Deep reactive ion etching was employed to construct PTFE nanowires aligned on the surface. Isopropyl and deionized water were used to clean 50 μm thick PTFE films, which were then dried with nitrogen. During the etching process, DC sputtering was used on the surface of the PTFE film as a mask to deposit Au particles for 45 s. Next, a gas mixture containing O_2_, CF_4_, and Ar was introduced to the inductively coupled plasma chamber, at corresponding flow rates of 10.0, 30.0, and 15.0 sccm, respectively. The nanowire structure was obtained on the surface by etching the PTFE film for 15 s. The high-density plasma was generated by a 500 W power source while the plasma ions were triggered by another 160 W.

### Fabrication of the u-TENG

The u-TENG fabrication procedure was adapted, with modifications, from [34]. The back electrode was formed by depositing a Cu layer on the unmodified surface of a PTFE film via magnetron sputtering. A poly(dimethylsiloxane) (PDMS)-coated PTFE film was mounted onto a poly(ethylene terephthalate) (PET) substrate. The Kapton film was fixed with a row of steel rods and heated, for 4 h in an oven at 100 °C, to achieve a wavy configuration. Copper foils were deposited onto both sides of the Kapton film by electron beam evaporation to form the wave-shaped electrode. The lead was connected to the electrode as an output terminal.

### Characterization and electrical measurement of the u-TENG

Field-emission scanning electron microscopy (FESEM, Hitachi SU-8020) was used to characterize the surface morphology of the modified PTFE film. A Stanford Research Systems equipment was used to measure the output performance of the u-TENG. Voltage and current were recorded using a Keithley 6514 electrometer.

## Results and Discussion

**Figure 1:**
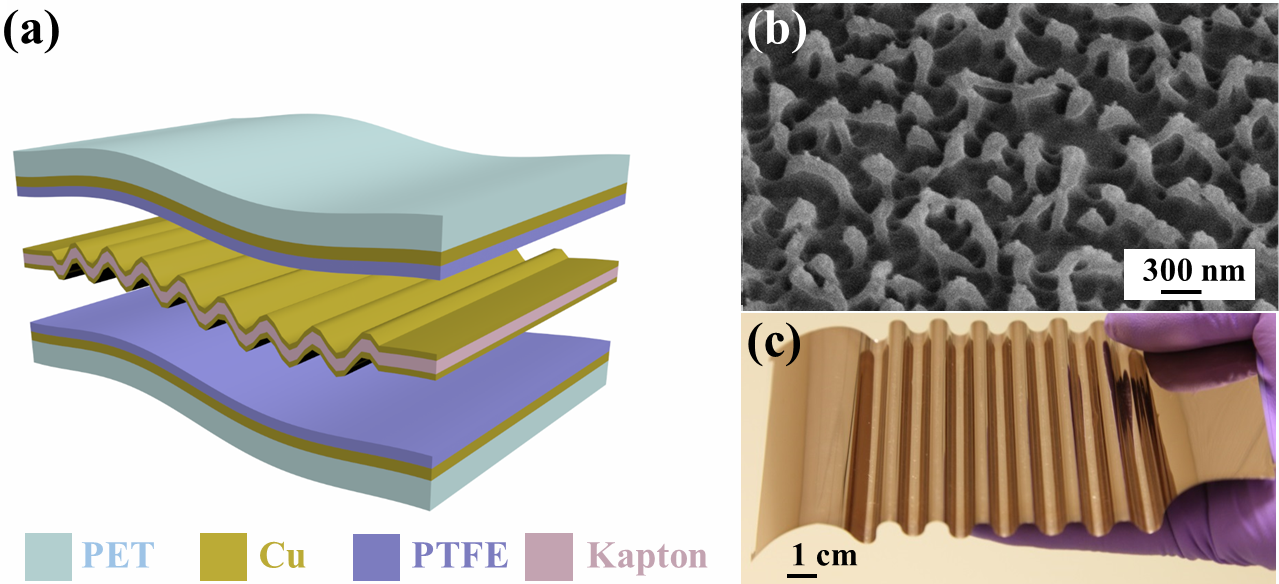
A flexible undulated electrode-based triboelectric nanogenerator. (a) Schematic diagram of the fabricated u-TENG. (b) FESEM image of an inductively coupled plasma-etched PTFE film. (c) Representative picture of the as-prepared undulated electrode.

The structured diagram of the fabricated undulated electrode-based triboelectric nanogenerator is shown in Figure 1a. This nanogenerator is composed of PET substrates, nanostructured PTFE thin films coated with copper foils (back electrodes), and an elastic undulated electrode in between. The internal wave-shaped electrode is obtained by depositing copper layers onto both sides of the wave-shaped Kapton film, as displayed in Figure 1a. Due to their huge difference in electron affinity [52], copper and PTFE were selected as the contact materials. The electrons flow between the undulated electrode and the planar electrode since the external mechanical impact repeatedly compresses the elastic corrugated electrode, shortening the distance between the PTFE film the undulated electrode. Through deep reactive ion etching, polymer nanowires (average diameter of ≈150 nm and length values ranging from 410 to 680 nm) were created to vertically align on the surface of the PTFE film, as shown in Figure 1b. This modification on the PTFE surface not only enhances the effective contact area with the undulated electrode but also promotes the triboelectric charge density on the friction surface. The prepared u-TENGs are flexible, rugged, light, and small devices, as revealed in Figure 1c. It is worth noting that the application of the undulated electrode structure in this work is totally different from its application in the previously reported work [34]. In the latter, the undulated elastic electrode configuration was utilized to harvest the impact from the water waves together with a solid–liquid interface generator to collect the electrostatic energy from the water body. In this manuscript, however, this design enables for an efficient approach for harvesting energy from the walking flow and for generating a self-powered pedestrian tracking system.

**Figure 2:**
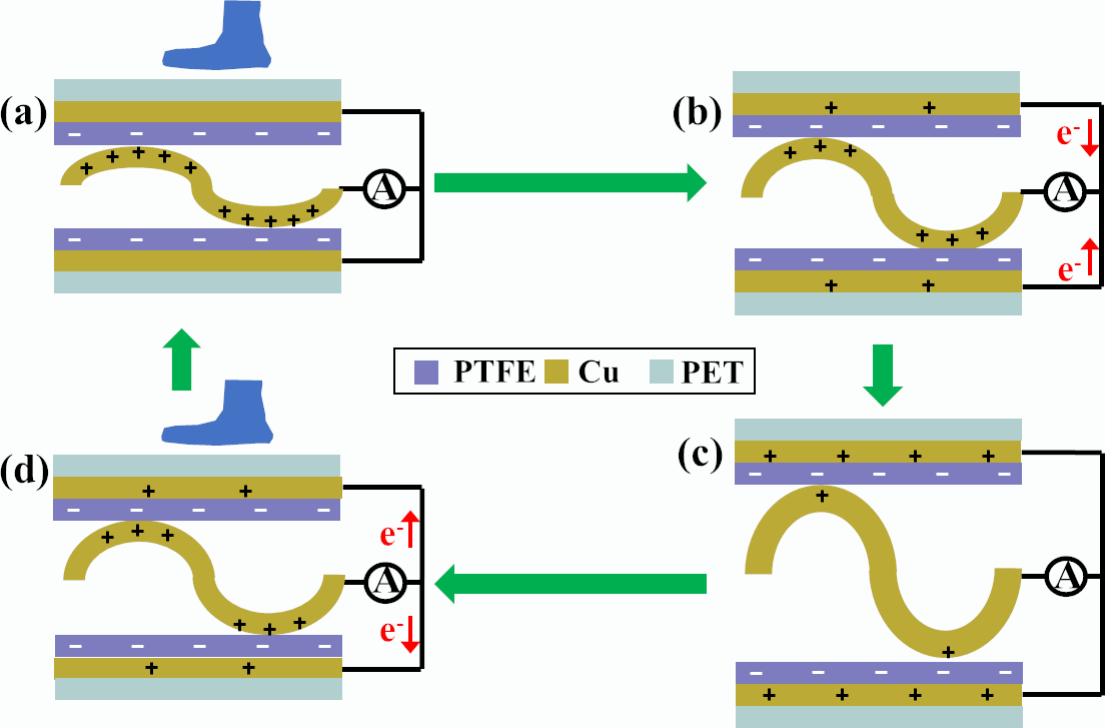
The working mechanism of the fabricated u-TENG in response to a stepping force. (a) The PTFE film and the undulated Cu electrode are brought into intimate contact by an external applied force. (b) When the force is released, free electrons flow from the Cu planar electrode to the undulated electrode. (c) The u-TENG reverts back to its unloaded position. (d) An external force compresses the u-TENG, driving the electrons move from the undulated electrode to the planar electrode.

The working principle of the u-TENG relies on the coupling between triboelectrification and electrostatic induction [34], as shown in Figure 2. The application and release of a stepping force during walking induces a periodical change between contact and separation the PTFE films and the undulated copper foils. This leads to the conversion of mechanical energy into electricity, as sketched in Figure 2.

**Figure 3:**
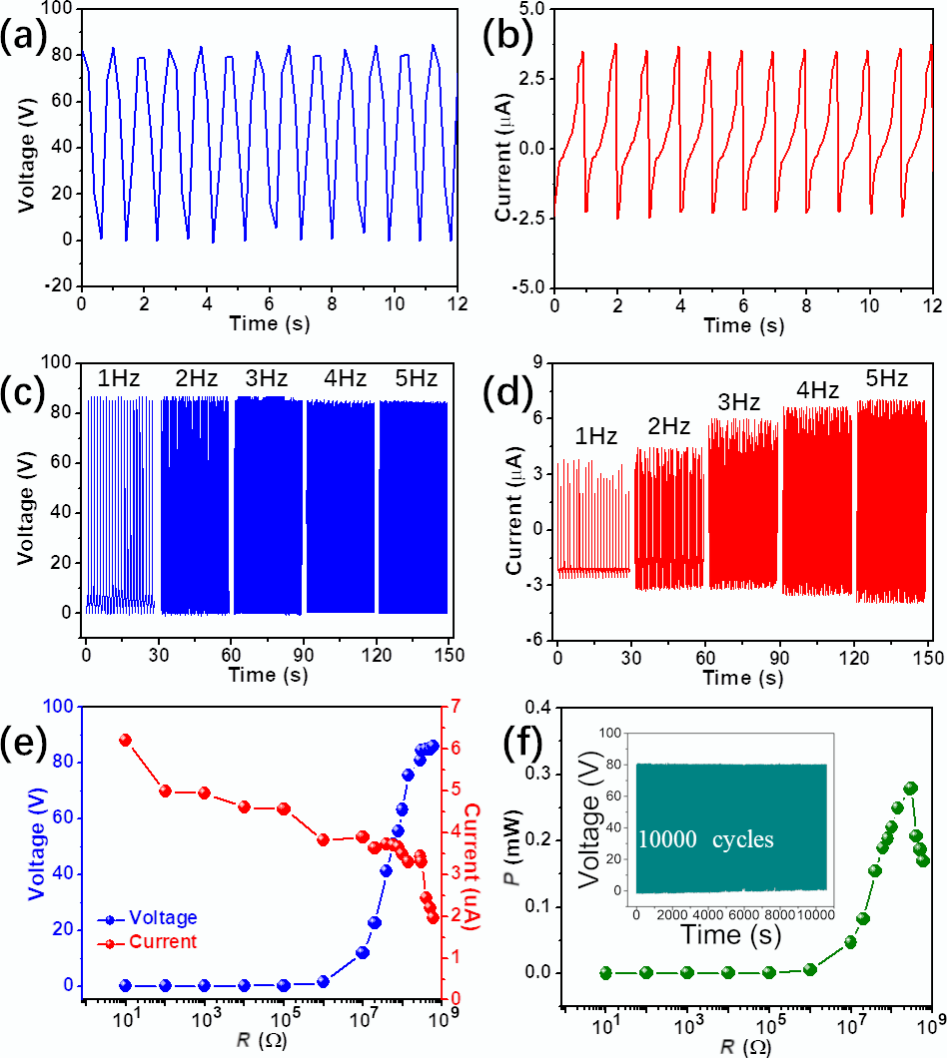
Electrical measurement results of the u-TENG. Open-circuit voltage (a) and short-circuit current (b) of the prepared u-TENG at an impact frequency of 1 Hz. Open-circuit voltage (c) and short-circuit current (d) of the prepared u-TENG under different frequencies ranging from 1 to 5 Hz. (e) Dependence of the output voltage and current on the external loading resistance. (f) Plot of the power density as a function of the loading resistance; inset: long-term stability.

To quantitatively characterize the u-TENG output performance, a linear motor was used to apply a periodical and controllable impact with tunable amplitude, frequency, and force. Triggered by an impact of 500 N at a frequency of 1 Hz, the as-prepared u-TENG delivered an output voltage of 86.0 V and an output current of 6.2 μA, as revealed in Figure 3a and Figure 3b, respectively. Output voltage and current both exhibit periodic behavior under repeated impact. In a frequency region of less than 5 Hz the output performance of the u-TENG is significantly higher than that of the EMG. Consequently, the frequency-dependent output behavior of the prepared u-TENG was investigated, as shown in Figure 3c and Figure 3d. As expected, the output current increased with an increasing frequency, while the output voltage remained almost constant. At an impact frequency of 5 Hz, the open-circuit voltage was 86.0 V and the short-circuit current was 10.8 µA. The impedance dependence of the fabricated u-TENG is shown in Figure 3e. The maximum output power of 0.279 mW can be observed at a loading resistance of 300 MΩ in Figure 3f. A long-term stability test did not exhibit a noticeable response decline after 10000 cycles (inset of Figure 3f), which indicates the robustness and repeatability of the prepared device.

**Figure 4:**
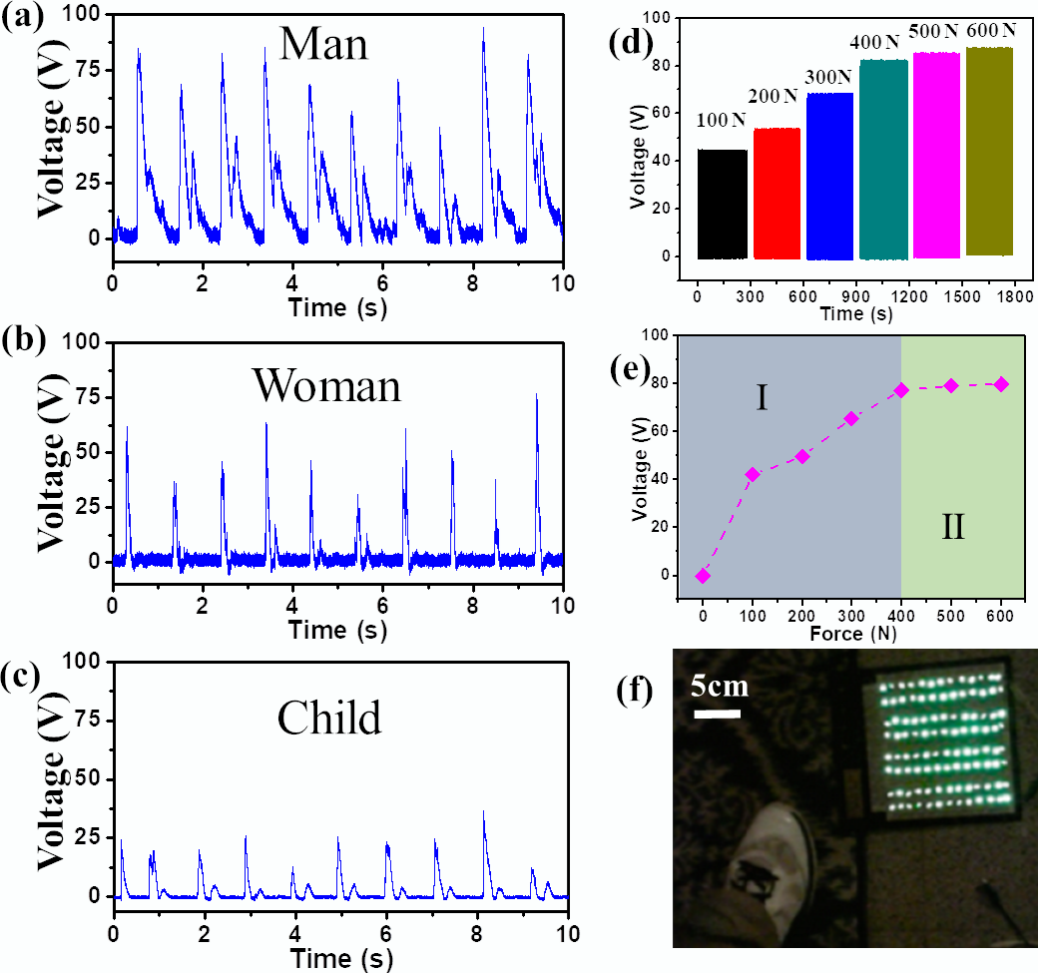
The ability of the u-TENG to harvesting energy from human walking. The electric output profile of a man (a), a woman (b), and a child (c). (d) The output voltage of u-TENG under different stress values. (e) The dependence of the output voltage on the impact force. (f) 110 LED bulbs are lit by stepping on a u-TENG.

To verify the capacity of harvesting human walking energy, the as-fabricated u-TENG was mounted as a floorboard to collect the mechanical energy from footsteps. The real-time dynamic signal profile of the output voltage for an adult man, an adult woman, and a child, respectively, was plotted in Figure 4a–c. These profiles show that our device is able to harvest a variety of walking motions from a wide range of people. It is evident that the walking steps can yield an electrical output. The output intensity is proportional to the pedestrian weight. This is because a more intense impact force causes a larger deformation of the undulated electrode, which contributes to larger separation (*d*) and thus a stronger output voltage.

To quantitatively study the dependence of the electric output on the force, the output voltage under various force values was plotted in Figure 4d. With an increasing force, the output voltage increases linearly at first and then it gradually saturates (Figure 4e). This can be explained by the combination of elastic and inelastic deformations. Under a relatively low external force, the induced elastic deformation increases almost linearly with the applied external force (region I). When the applied force increases, the elastic deformation is gradually converted into an inelastic deformation, which makes it harder to trigger a further deformation under an increasing force. According to the theoretical analysis of the TENG [53],

[1] $$V_{\text{OC}}=\frac{\sigma \cdot d}{\varepsilon_{0}\cdot \varepsilon_{\text{r}}},$$

where *σ* is the surface triboelectric charge density, *d* is the interlayer distance, ε_0_ is the vacuum permittivity, and ε_r_ is the relative permittivity of the PTFE layer. The standstill deformation gives rise to the saturated output voltage in the inelastic deformation region (region II), as shown in Figure 4e. It was noted during the testing that the device was smaller than the sole of the feet of the participants. Therefore, only a part of the body weight was compressing the device. Hence, the applied force was smaller than the weight of a given participant. As a result, this feature leads to a larger difference in the output signal between a man (77 kg) and a woman (46 kg) according to Figure 4d.

To further demonstrate the device performance, 110 LED bulbs in series were linked to the as-fabricated u-TENG. As displayed in Figure 4f, all LED bulbs were simultaneously lit by a simple foot step, indicating the potential of the u-TENG as a sustainable power source.

**Figure 5:**
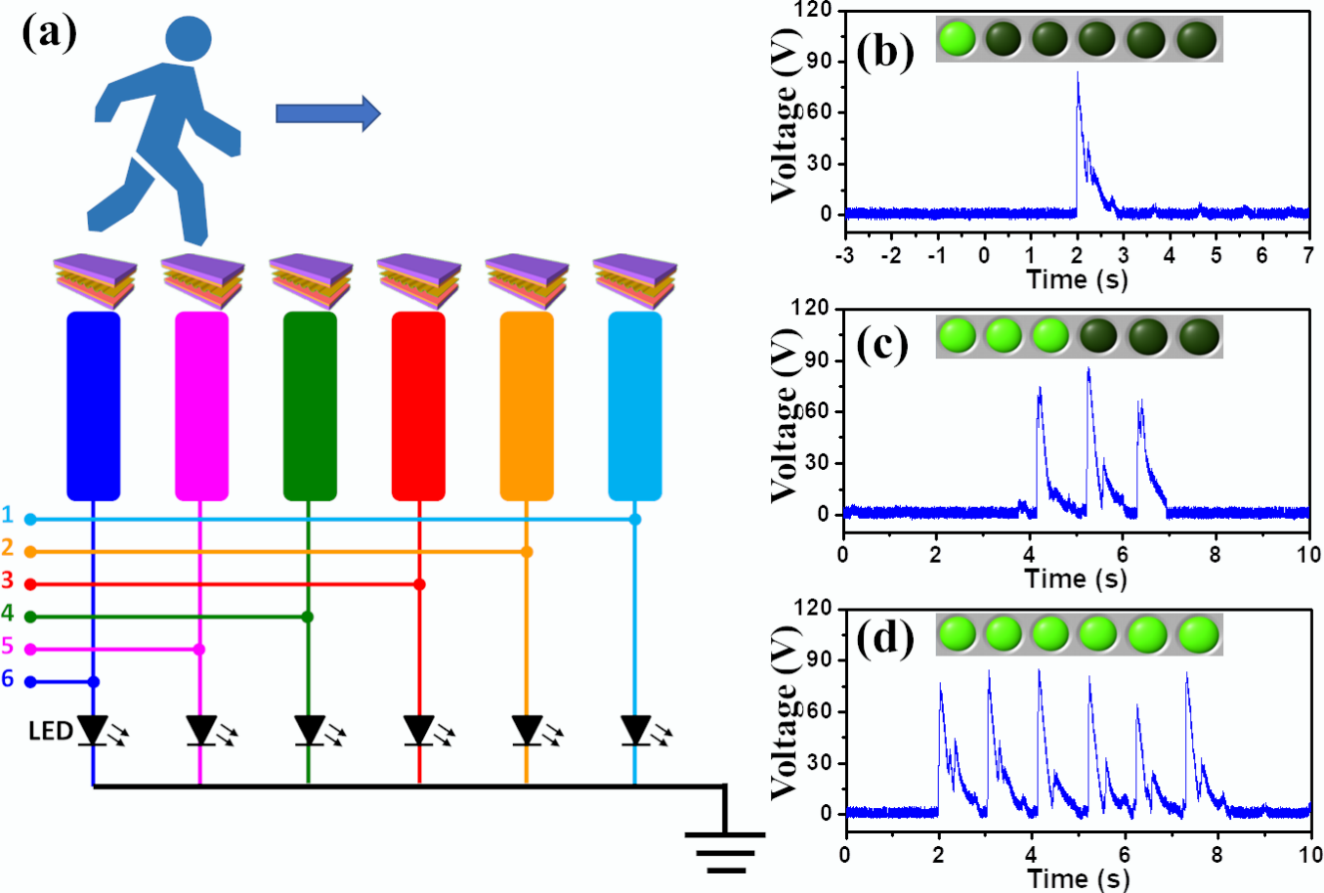
Self-powered u-TENG-based location-tracking system. (a) Circuit diagram of the fabricated u-TENG-based tracking system. (b–d) Measured output voltage and real-time location mapping when the pedestrian arrives at the (b) first, (c) third, and (d) sixth electrode.

To testify the feasibility of the fabricated u-TENG for a location-tracking system, six as-prepared u-TENGs were integrated into six floor blocks along a public aisle in order to detect and map the real-time position of a pedestrian, as shown in Figure 5a. Six channels were assigned via Labview to the corresponding six floorboards for real-time tracking. The output voltages from the six channels were recorded and instantly triggered the corresponding indicator on a monitor. When the pedestrian reached the first floorboard, a relevant voltage peak of 86.3 V was detected, as shown in Figure 5b. The signal triggered the first positioning indicator on a monitor, revealing that the pedestrian had just arrived at the first floorboard. The electric output also lit the LED bulb that was connected to the stepped floorboard and it emitted a visible light sign that indicated the instantaneous location of the pedestrian. It is important to mention that the LED light emission was powered by the triboelectric generator driven by the walking movements without any electric power source. Figure 5c and Figure 5d show the output voltage profile and the real-time mapping results when the pedestrian passes the third and the sixth floorboard, respectively. Therefore, the positional information of the pedestrian can be immediately displayed and directly observed, enabling pedestrian volume counting and passenger tracing. This design could be utilized in low-traffic areas or public passages at night to provide an alternative for energy harvesting and conservation in modern cities.

## Conclusion

In sum, we demonstrated that an undulated electrode-based TENG can be used to harvest walking energy from pedestrians. Triggered by the steps of human walking, an open-circuit voltage of 86 V and a short-circuit current of 6.2 μA were obtained from the as-fabricated u-TENG, which can continuously light up 110 LED bulbs. Moreover, the u-TENG can also harvest the mechanical energy from a variety of pedestrians, including men, women and children. In addition, integrated with six sensing channels along a public aisle, a self-powered location-tracking system was constructed for pedestrian volume counting and passenger tracing. This work paves the way for the application of triboelectric sensors in intelligent cities and for low-carbon transportation alternatives.
